# Development and Implementation of an IoT-Enabled Smart Poultry Slaughtering System Using Dynamic Object Tracking and Recognition

**DOI:** 10.3390/s25165028

**Published:** 2025-08-13

**Authors:** Hao-Ting Lin, Suhendra

**Affiliations:** 1 Department of Bio-Industrial Mechatronics Engineering, National Chung Hsing University, 145 Xingda Rd., South Dist., Taichung City 402, Taiwan; 2 Departement of International Doctoral Program in Agriculture, National Chung Hsing University, 145 Xingda Rd., South Dist., Taichung City 402, Taiwan; d112030206@mail.nchu.edu.tw; 3 Departement of Agro-Industrial Technology, Universitas Bengkulu, WR. Supratman St., Muara Bangka Hulu Dist., Bengkulu 38371, Indonesia

**Keywords:** IoT, poultry slaughter, image recognition, dynamic tracking object

## Abstract

**Highlights:**

**What are the main findings?**

- Developed an IoT-enabled, AI-driven humane poultry slaughtering system for red-feathered Taiwan chickens using YOLO-v4-based dynamic object tracking. Also, the system was successfully implemented in a real slaughterhouse, demonstrating a practical AI application in humane poultry slaughtering.
- Achieved 94% mean average precision (mAP) with a real-time detection speed of 39 fps, enabling accurate distinction between stunned and unstunned chickens using the YOLO-v4 model and image enhancement.

**What is the implication of the main finding?**

- Provides a scalable, automation-ready solution for enhancing animal welfare compliance, reducing labor dependency, and improving hygiene standards in poultry slaughterhouses.
- Demonstrates the viability of integrating deep learning and sensor networks into closed-loop, IoT-monitored smart agriculture systems for real-time decision making.

**Abstract:**

With growing global attention on animal welfare and food safety, humane and efficient slaughtering methods in the poultry industry are in increasing demand. Traditional manual inspection methods for stunning broilers need significant expertise. Additionally, most studies on electrical stunning focus on white broilers, whose optimal stunning conditions are not suitable for red-feathered Taiwan chickens. This study aimed to implement a smart, safe, and humane slaughtering system designed to enhance animal welfare and integrate an IoT-enabled vision system into slaughter operations for red-feathered Taiwan chickens. The system enables real-time monitoring and smart management of the poultry stunning process using image technologies for dynamic object tracking recognition. Focusing on red-feathered Taiwan chickens, the system applies dynamic tracking objects with chicken morphology feature extraction based on the YOLO-v4 model to accurately identify stunned and unstunned chickens, ensuring compliance with animal welfare principles and improving the overall efficiency and hygiene of poultry processing. In this study, the dynamic tracking object recognition system comprises object morphology feature detection and motion prediction for red-feathered Taiwan chickens during the slaughtering process. Images are firsthand data from the slaughterhouse. To enhance model performance, image amplification techniques are integrated into the model training process. In parallel, the system architecture integrates IoT-enabled modules to support real-time monitoring, sensor-based classification, and cloud-compatible decisions based on collections of visual data. Prior to image amplification, the YOLO-v4 model achieved an average precision (AP) of 83% for identifying unstunned chickens and 96% for identifying stunned chickens. After image amplification, AP improved significantly to 89% and 99%, respectively. The model achieved and deployed a mean average precision (mAP) of 94% at an IoU threshold of 0.75 and processed images at 39 frames per second, demonstrating its suitability for IoT-enabled real-time dynamic tracking object recognition in a real slaughterhouse environment. Furthermore, the YOLO-v4 model for poultry slaughtering recognition in transient stability, as measured by training loss and validation loss, outperforms the YOLO-X model in this study. Overall, this smart slaughtering system represents a practical and scalable application of AI in the poultry industry.

## 1. Introduction

The growing global population has led to a continual increase in demand for high-quality poultry protein. Consequently, conventional slaughtering practices face the challenge of ensuring that strict hygiene standards are met at every step of the process to prevent meat contamination and minimize food safety risks [1,2]. At the same time, social awareness regarding animal welfare has increased; people are increasingly realizing that raising production efficiency should not be at the expense of animal welfare [3,4]. In addition, regulations have been enacted to safeguard the welfare of poultry during the raising, transportation, and slaughter processes [5,6]. According to Taiwan’s Animal and Plant Health Inspection Agency, the annual number of broilers slaughtered in Taiwan has increased consistently in recent years. Smart production technologies are an integral component of improving production efficiency while protecting animal welfare; in particular, image recognition technology has offered new options for the poultry slaughtering process. Conventional slaughter practices can be rife with problems such as improper operations, animal suffering, and low production efficiency. Real-time monitoring and smart management achieved through image recognition technology can improve the quality and efficiency of poultry slaughtering [7,8,9]. Because of increased social attention to food safety and animal welfare, poultry slaughterhouses require intelligent, scientific, and humane solutions. Recent advances in poultry processing have demonstrated that IoT-enabled platforms and cloud-integrated monitoring can significantly improve the transparency, responsiveness, and efficiency of slaughterhouse operations [10,11,12]. IoT-based sensor networks have been proven effective in detecting stress, monitoring poultry welfare, and enhancing slaughter [13,14,15]. In 2024, Anita [16] reviewed and outlined IoT architecture, enabling technologies, market potential, and simulators, offering a comprehensive foundation for researchers to address existing issues and drive future advancements in IoT solutions.

## 2. Literature Review

A stress response in an animal before slaughter can induce muscle metabolism, which affects the quality of the meat. The effects of transport-induced stress on blood metabolism, glycolytic potential, and meat quality in broilers have been evaluated [17]. Based on their results, long-term transport caused a decrease in muscle fibers and an increase in their density, meaning that texture was affected. Out of forty 180-day-old female black bone chickens raised under identical conditions and slaughtered by the same butcher, the quality of meat of broilers that had been stunned was compared with that of broilers that had not been stunned prior to slaughter [18]. Based on the results, the carcasses of the broilers that were stunned prior to slaughter had lower shear force values and less bleeding, resulting in higher quality meat compared with unstunned broilers. Forty-two-day-old broilers, each weighing 2.4 ± 0.8 kg, were stunned with pulsed direct currents (DCs) of 5 V, 15 V, 25 V, 35 V, and 45 V at 700 Hz to examine the effects of different voltages on characteristics such as meat quality and protein solubility. The results indicated that 5 V, 35 V, and 45 V resulted in the most damage to the quality of the chicken meat, and 5 V and 45 V accelerated the postmortem pH decline rate, diminishing the water holding capacity and shear force [19].

With advancements in agricultural technology, the poultry farming industry has progressed toward higher efficiency and smarter developments. Image recognition technologies have provided poultry farmers with many innovative solutions that improve the cost, precision, and efficiency of poultry farming. Through model training, image recognition can continuously monitor poultry behaviors, particularly their eating and resting habits, as well as their health status. Analysis of potential diseases enables poultry farmers to apply corrective measures immediately in order to minimize the spread of disease and loss of life. Intelligent management of poultry can promote production efficiencies and economic benefits. Smart poultry systems also benefit from digital twin modeling, which enables the synchronized simulation of physical environments in parallel with edge and fog computing frameworks to ensure low-latency responses and system dependability [20,21]. The YOLO-v4 was proposed for a CSPDarknet53 architecture combining Cross-Stage Partial connections (CSP) and Darknet-53 to markedly improve the perceptiveness and accuracy of the network [22]. In 2021, Liu et al. [23] proposed the YOLO-v4 algorithm to identify images of dead chickens with a chicken removal system. The designed system achieved a precision of 95.24%, and dead chickens were successfully moved to the storage cache of a chicken removal system. In 2023, Bist et al. [24] proposed YOLO-v5 and YOLO-v6 to test and compare their ability to identify dead birds. This study verified that litter and feather coverage and camera height affected the precision of the image recognition model. When the camera was placed in poultry housing with no feather coverage, the YOLO-v5 model achieved a mean average precision (mAP) @0.50 score of 99.4% and a precision score of 98.4%. The results indicated that YOLO-v5 outperformed YOLO-v6 in precision, recall, processing speed, and training duration. In 2025, Shen et al. [25] proposed point-level fusion of LiDAR and RGB images for enriched semantic–geometric representation. Additionally, Efficient Channel Attention is designed to emphasize key features for small objects. In the KITTI dataset, small object detection and orientation estimation can be significantly improved, enhancing object tracking and decision-making in dynamic environments. In 2025, Shams et al. [26] introduced a camera-based method using morphological changes across growth stages. The method applies YOLO-v8, a deep learning segmentation technique, for accurate broiler detection. The model achieves a mean average precision of 0.829 across IoU thresholds from 50% to 95%, enabling efficient, non-intrusive, and reliable broiler weight estimation without the need for direct measurement. In 2025, Park et al. [27] proposed image augmentation techniques to enhance non-PPE detection on construction sites using deep learning. Eight augmentation methods were tested, revealing that their impact on accuracy is task- and architecture-dependent. Results showed that tailored augmentation strategies improve performance with potential applicability beyond non-PPE detection, such as identifying equipment or hazardous behaviors in construction environments. In 2024, Wang [28] proposed nine image augmentation techniques for detecting windows and their states from building façade images using various deep learning models. Results showed that augmentation effectiveness varies by architecture and metric, and combining methods is not always optimal. Tailored augmentation strategies are essential, with potential applications extending to tasks such as window type detection and building age classification. In 2025, Jiang et al. [29] proposed a real-time poultry monitoring system featuring an attention-enhanced YOLO-Chicken algorithm and BoT-SORT tracking. YOLO-Chicken improves detection accuracy and speed, outperforming the original YOLOv8 by over 1.35%. It also exceeds the performance of classical models such as Faster R-CNN, RetinaNet, and other YOLO variants in both accuracy and efficiency, offering a reliable and effective solution for automated poultry management and monitoring in real-time applications.

While YOLO-based object detection models have shown significant promise in automating poultry monitoring and management, as well as in construction, the deployment of YOLO-based systems requires careful calibration, specialized datasets, and adequate computational infrastructure, which may limit scalability and applicability across diverse poultry farming contexts. Additionally, while newer versions of YOLO offer superior usability, flexibility, and accuracy—especially in detecting small or complex objects—YOLO-v4 remains relevant in specific contexts due to its performance efficiency, legacy integration, and solid accuracy. The choice depends on the balance between available resources, required precision, and system compatibility. The objective of this study was to build a smart and humane poultry slaughtering system for monitoring, determining, and managing the electrical stunning of red-feathered Taiwan chickens using dynamic tracking object recognition. Brainwaves and manual identification were adopted to identify stunned and unstunned red-feathered Taiwan chickens after electrical stunning in order to train the image recognition model and thus achieve automated monitoring, identification, and interpretation of chickens during the electrical stunning and slaughter processes. To ensure remote operability and responsive automation, the system architecture incorporates IoT-enabled components for sensor integration, real-time data acquisition, and cloud-compatible monitoring throughout the stunning process. YOLO-v4 is considered suitable for AI slaughtering applications due to its high accuracy, real-time processing speed, and strong generalization capabilities. Its CSPDarknet53 backbone improves network accuracy and perceptiveness, while the integration of Spatial Pyramid Pooling (SPP) and Path Aggregation Network (PANet) enables effective detection of poultry at varying scales and positions along the slaughter line. Thus, stunned and unstunned chickens can be reliably identified in dynamic real-world conditions. Additionally, image enhancement techniques (e.g., cropping, scaling, and rotation) can be supported in the model, improving robustness and adaptability to varied slaughterhouse environments. Given its strengths in immediacy, accuracy, and efficiency, YOLO-v4 is well-suited for real-time, humane poultry monitoring in automated slaughter systems. Additionally, the system is expected to help prevent infringements related to the welfare of red-feathered chickens during the electrical stunning process in the event of insufficient electrical charge (possibly resulting in the chicken remaining conscious while being slaughtered) or excessive electrical charge killing the chicken immediately. The primary goal of this study was, therefore, to ensure that the animal welfare rights of Taiwan chickens could be upheld during the slaughtering process. The secondary goal of this study was to increase the level of intelligence in local poultry slaughtering processes. Replacing conventional and manual post-stunning detection techniques with computer-based image recognition techniques could help improve the efficiency and accuracy of chicken stunning. It could also enable the immediate discovery and handling of critical situations when they occur, thereby reducing labor costs while increasing production line efficiency. By monitoring the stunning conditions of native chickens, stress responses induced by electrical stunning and even death due to excessive electric shock can be detected and corrected immediately, thereby improving the quality of the carcass. Furthermore, reducing personnel’s contact with the production line would likely improve hygiene standards and thus reduce the risk of infection.

## 3. Materials and Methods

### 3.1. Smart Humane Poultry Slaughter System Processes

**Figure 1:**
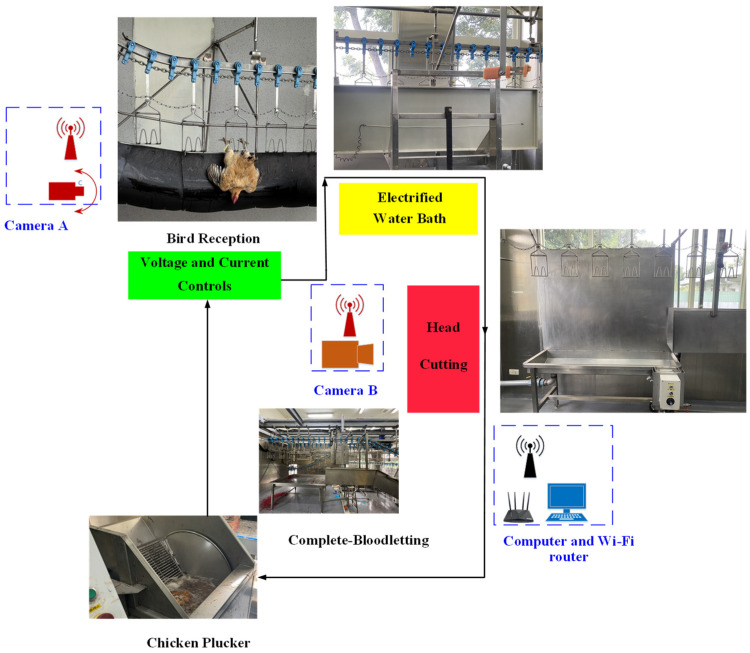
Schematic of the smart humane poultry slaughter system.

The proposed system for the smart humane slaughter of red-feathered Taiwan chickens comprises bird reception, an electrified water bath, head cutting, voltage and current controls, chicken plucking, Camera A (Xiaomi, China) capturing the bird reception section, Camera B (Xiaomi, China) capturing the bloodletting, a computer, and a wireless router (D-Link, Taiwan); see Figure 1 for a detailed schematic of the system. The approval animal number for this research is 111-008^R3^. The age of the red-feathered Taiwan chickens is 16 weeks. The slaughter process for the red-feathered Taiwan chickens is as follows:

1. During reception, red-feathered Taiwan chickens waiting to be slaughtered are hung by their feet from an overhead conveyor to be transported upside-down to the water bath for electrical stunning. Shackles ensure that the chickens are firmly secured to the overhead conveyor, preventing them from falling from the conveyor when immersed in the electrified water bath and damaging equipment or harming personnel. This design guarantees the safety of the slaughter process and maximizes work efficiency by securing the chickens’ feet to the overhead conveyor and confirming that the chickens’ bodies are in the proper position. This operational procedure ensures that the red-feathered Taiwan chickens move smoothly through the slaughter process while minimizing risks.
2. The red-feathered chickens are electrically stunned during the electrified water bath. More specifically, the chickens are shocked for 7 ± 0.6 s with a constant voltage DC to render them unconscious. The positive electrode of the constant voltage DC is connected to the water bath, and the negative electrode is connected to the overhead conveyor. This setup ensures that the red-feathered Taiwan chickens are fully stunned and prevents situations in which any chicken remains conscious because the electrical current did not completely pass through their body. In addition, this design reduces the risk of electrical shock to operators, thereby ensuring the safety of the slaughter process.
3. The head-cutting area is where slaughterers cut the throats of the red-feathered Taiwan chickens. After being stunned by the electrified water bath, the unconscious chickens are killed and bled in this area to complete the slaughter process. In this study, Camera B was positioned in this area to capture images of successfully and unsuccessfully stunned red-feathered Taiwan chickens; these images were subsequently used to train the identification model. The trained model was then used for real-time stunning identification to ensure that the treatment of the red-feathered chickens met animal welfare requirements prior to slaughter. In addition to images, brainwaves were also collected in the head-cutting area; these data can contribute to a deeper understanding of the physiological responses of red-feathered Taiwan chickens during the electrical stunning process and further guarantee proper stunning effect and animal welfare. To facilitate data collection and real-time monitoring, this study employed high-resolution cameras and a high-speed router to ensure that the collected images and data could be instantly and accurately transmitted to the computer via Wi-Fi. The collection and analysis of these data can help improve and optimize the electrical stunning process, ensuring that every red-feathered Taiwan chicken can be rendered properly unconscious before being slaughtered, thereby minimizing their suffering. In addition, these technological measures can enhance the safety and efficiency of the slaughter process, preventing risks to operators.
4. Red-feathered chickens that are confirmed to have no vital signs are transported by the overhead conveyor to the complete bloodletting area and continue to bleed during transport. All red-feathered chickens in this area are dead and are released automatically from the overhead conveyor onto a bench.
5. The slaughterers place the bled chickens into the chicken pluckers for feather removal. First, the carcasses of the red-feathered Taiwan chickens are scalded to warm them up and thus facilitate subsequent defeathering. The carcasses are continually tumbled in the pluckers to ensure that the red-feathered Taiwan chickens are sufficiently heated in the drum, which is padded with soft plastic columns. When a red-feathered Taiwan chicken carcass is dumped into the drum, the soft plastic columns collide with the carcass, and the force from the high-speed rotations plucks the feathers from the carcass. When feather removal is complete, the carcass is ejected from the plucker.

Following the final step, each red-feathered Taiwan chicken carcass goes through subsequent processes, including evisceration and packaging. Defeathering ensures the clean and attractive appearance of the carcass and improves the efficiency and quality of subsequent processing. The scaling and plucking durations of the plucker can be adjusted according to the broiler species.

### 3.2. Image Recognition System Equipment and Architecture

Training the model to identify stunned red-feathered Taiwan chickens requires a high-performance computer to enhance the training effect and training speed. The system must have stable operating performance and high-speed processing capabilities during image recognition and data storage in the slaughterhouse; hence, a computer with high-performance processors and high storage capacity was chosen in this study. Furthermore, to meet the processing demands for large amounts of data, the training and inference processes of the deep learning model were accelerated using a high-speed solid-state drive (SSD) and a powerful GPU. The central processing unit (CPU) was a high-performance multicore processor that ensured the rapid processing of complex computing tasks in multiple execution environments. In this study, the CPU was used to preprocess the training set by adjusting the image sizes and rotating and enhancing the images. Model evaluation and system management were performed simultaneously to ensure the high-performance operation of the image recognition model processes. In this study, the training process was accelerated by the GPU, whose large number of cores and parallel computing capabilities are particularly well-suited to the efficient execution of large-scale matrix operations and deep learning calculations, meaning that it can rapidly iterate models and optimize parameters. Random access memory (RAM) enables the efficient storage of and access to large quantities of data during the training of large-scale models. In this study, RAM enabled the storage of an entire batch of data in one action, ensuring that the training process was continuous and efficient. The hard drive was an SSD, which was used to read and write data rapidly and to minimize bottlenecks during data transmission. A large storage drive was used to store the training data in this study. The specifications of all the equipment used in this study are presented in Table 1.

This study required the collection of images of stunned red-feathered Taiwan chickens through cameras to train the identification model. Images of the slaughter line also needed to be captured by the cameras and transmitted to the computer via the Wi-Fi router to facilitate real-time image recognition in subsequent applications. As part of an IoT-based acquisition system, this data transmission infrastructure enables the integration of edge devices and wireless communication to support real-time recognition and feedback. Training data were collected from two different angles: Camera A was positioned 30 cm from the bird reception area and focused on capturing the appearance of the red-feathered Taiwan chickens before their immersion in the electrified water bath; Camera B was positioned 60 cm from the bloodletting area. Together, these two cameras captured images of the red-feathered Taiwan chickens from different angles before and after the chickens were electrically shocked, providing images for both labeling and training. Consequently, high-speed transfers and high-definition image quality were necessary in this study to guarantee the accuracy and timeliness of the data. To this end, high-resolution cameras were utilized to ensure that clear and detailed images could be captured to maximize the accuracy of the model. Moreover, selecting a Wi-Fi router that supports high-speed transfer enabled immediate feedback of images; the router employed in this study supports a typical broadband network and 4G SIM cards, enabling it to switch between the two and thus preventing connectivity problems from hindering system operations while satisfying real-time identification requirements. These components collectively form the edge layer of the proposed IoT architecture, ensuring that the stunning process is continuously monitored and analyzed with minimal latency. The proper use of the aforementioned equipment is expected to significantly enhance the training of the stunned red-feathered Taiwan chicken identification model, ensuring that each chicken is slaughtered in compliance with animal welfare standards and minimizing stress and pain during the slaughter process. Beyond image-based detection, the integration of IoT simulation and remote monitoring capabilities enhances system intelligence and supports closed-loop animal welfare verification.

### 3.3. IoT Integration and Digital Twin Simulation

For an image sensor sampling model based on a sensor node, the discretization of continuous images based on the center of the $\left(i,\;j\right)-th\;pixel:\left(x_{i},y_{j}\right)$ for a digital grid can be represented as follows:

(1) $$I\left[i,j\right]=I\left(x_{i},y_{j}\right)$$

where $i=1,\ldots ,\;N_{x},\;j=1,\ldots ,\;N_{y}$, $N_{x}\times N_{y}$ is the resolution of the image sensor. With IoT edge processing, Gaussian noise will affect image transmissions due to packet corruption. Thus, the actual values of the sensor records corrupted by the Gaussian noise can be described as follows:

(2) $$I_{measured}\left[i,j\right]=I\left[i,j\right]+n_{G}\left[i,j\right]$$

(3) $$\hat{I}=P(I_{measured}\left[i,j\right])$$

where $n_{G}\left[i,j\right]\sim N(0,\;\sigma^{2})$, $\sigma^{2}$ is the variance of the noise, $\hat{I}$ is the IoT edge processing image result, and P is the processing function.

**Figure 2:**
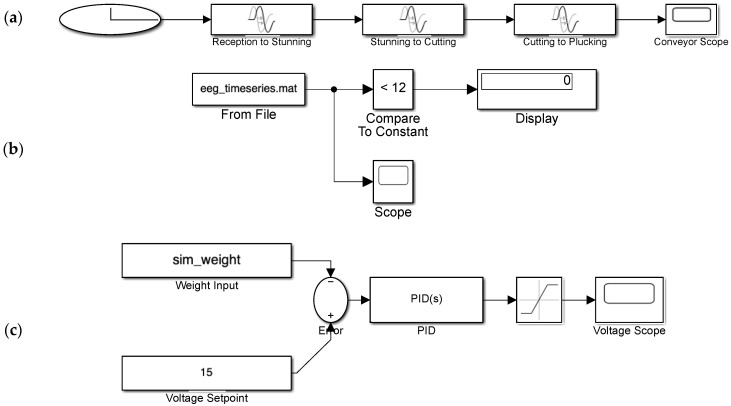
(**a**) Block diagram of the virtual poultry slaughtering process, (**b**) EEG-based stunning detection logic in Simulink, and (**c**) PID-based voltage control system structure.

To complement the image recognition component, an IoT-based simulation framework was developed to represent a digital twin of the poultry slaughtering process. Using MATLAB/Simulink (R2025a), a virtual conveyor model was built to simulate time-dependent transitions between four key processing stages: reception, stunning, cutting, and plucking. Each stage was modeled with a defined transport delay to reflect the realistic movement of poultry along the production line. This timing simulation, visualized via a stage-index signal (Figure 2a), enables stage-aware process monitoring in real time. In parallel, a logic-based EEG stunning detection subsystem was implemented to assess the consciousness state of chickens using threshold comparison. Dynamic EEG signals were processed and compared to a fixed threshold value of 12 mV. The output of this comparison generates a binary classification “stunned” or “not stunned”, which can be transmitted to IoT platforms by the MQTT-enabled dashboard for remote monitoring and decision support (Figure 2b). Such a system aligns with best practices in smart livestock architectures, where IoT-enabled sensors, data processing units, and cloud connectivity enable continuous monitoring and responsive control [30]. To ensure robustness and interoperability, data transmission in this system is modeled according to established frameworks for structured IoT data pipelines in smart farming [31].

Furthermore, a PID-based voltage control subsystem was developed to simulate adaptive control of stunning voltage based on variations in chicken weight. The simulated weight is compared with a setpoint voltage, and the resulting error is regulated by a PID controller to ensure that the voltage remains within an effective and safe stunning range. The block diagram of this control structure is illustrated in Figure 2c, providing a foundation for future closed-loop IoT-controlled stunning systems.

### 3.4. Building the Stunned Red-Feathered Taiwan Chicken Dataset

To build an accurate identification model, data from 200 red-feathered Taiwan chickens—including brainwaves, images, weight, and voltages—were collected to determine whether the stunned chickens had been successfully rendered unconscious. Brainwaves were used to accurately determine the current status of the red-feathered Taiwan chickens while judging the training data. In this study, brainwaves were divided into three intervals: 10–20 s (P1), 20–30 s (P2), and 30–40 s (P3). Differences in brainwave energy values before and after electric shock treatment were used to determine whether each red-feathered Taiwan chicken had been successfully stunned: If the post-stunning brainwave energy value was 10% of the pre-stunning value or lower, the chicken was deemed to have been successfully stunned. When selecting the training dataset, to prevent the model from being trained on erroneous data, judgments were made on the basis of both brainwaves and visual determinations; simultaneous judgments could enhance the accuracy of the stunned red-feathered Taiwan chicken dataset. To validate and label the data, the consciousness of red-feathered Taiwan chicken was divided into four scenarios. Table 2 presents the brainwave data sheets of electric shocks administered to red-feathered Taiwan chickens, including the four scenarios. Table 3 presents the visual criteria for each red-feathered Taiwan chicken, including raising its head, opening its eyes, moving its wings, its weight, and the voltage.

**Figure 3:**
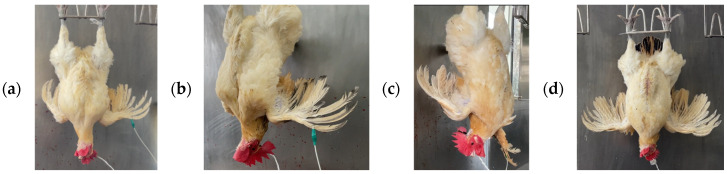
Red-feathered Taiwan chickens post-stunning: (**a**) deemed unconscious by sight and brainwaves, (**b**) deemed conscious by sight but unconscious by brainwaves, (**c**) deemed conscious by sight and brainwaves, and (**d**) deemed unconscious by sight but conscious by brainwaves.

Scenario 1: The chicken is determined by sight and by brainwaves to have been stunned; Figure 3a depicts a red-feathered Taiwan chicken in this state.

Scenario 2: The chicken is visually determined not to have been stunned, yet the brainwaves indicate unconsciousness; see Figure 3b.

Scenario 3: The chicken is determined by sight and by brainwaves not to have been stunned; see Figure 3c.

Scenario 4: The chicken is visually determined to have been stunned, yet the brainwaves indicate consciousness; see Figure 3d.

### 3.5. Dynamic Tracking Object Recognition for the YOLO-v4 Red-Feathered Taiwan Chicken Image Recognition Model

**Figure 4:**
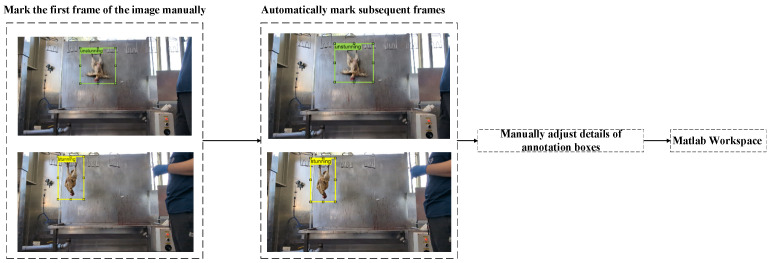
Dynamic tracking of the labeling process.

The YOLO-v4 image recognition model is a supervised learning algorithm that requires labeled data for model training. These labeled data include the position, size, shape, and attributes of the target object within the overall image, as well as the category to which the target belongs. In this study, the target categories are divided into two: red-feathered native chicken hens that have been stunned by electric shock, and red-feathered native chicken hens that have not been stunned. The label file and its corresponding image file are generated through the above-mentioned annotation information and provided to the image recognition model for training. For this study, the video labeler and image labeler in the MATLAB image processing and computer vision toolbox were adopted for labeling. Figure 4 shows the dynamic tracking for the labeling process.

The YOLO-v4 backbone utilized in this study was constructed using Darknet, an open-source neural network framework primarily used in machine vision and object detection tasks. Additionally, the YOLO-v4 model employed was constructed using a CSPDarknet-53 framework, which improves on the original Darknet-53 framework by incorporating the cross-stage partial network to enhance the model’s learning ability and computing speed. The YOLO-v4 model with object detection on input images, producing bounding boxes, can be described as follows:

(4) $$D_{t}={\left\{(b_{i}^{t},s_{i}^{t},\;c_{i}^{t})\right\}}_{i=1}^{N_{t}}\;$$

where $b_{i}^{t}=(x_{i}^{t},y_{i}^{t},w_{i}^{t},h_{i}^{t})$ is the set of image coordinates (center *x*, center *y,* width, height), $s_{i}^{t}\in [0,1]$ is the confidence score, $c_{i}^{t}$ is the object class, and $N_{t}$ is the number of detections at time t. Let each tracked object $o_{k}$ have a motion state:

(5) $$x_{k}^{t}={\left[u_{k}^{t}\;v_{k}^{t}\;{\dot{u}}_{k}^{t}\;{\dot{v}}_{k}^{t}\;w_{k}^{t}\;h_{k}^{t}\right]}^{T}$$

where ($u_{k}^{t},v_{k}^{t}$) is the position of the center of the frame, $\left({\dot{u}}_{k}^{t},\;{\dot{v}}_{k}^{t}\right)$ is the velocity of the frame’s center, $w_{k}^{t}$ is the width of the bounding box, and $h_{k}^{t}$ is the height of the bounding box. For IoT-enabled dynamic object tracking, Equation (5) can be described as follows:

(6) $$x_{k}^{t}={\hat{x}}_{k}^{t}+K_{t}(z_{k}^{t}-H{\hat{x}}_{k}^{t})$$

where $z_{k}^{t}$ is the matched detection state, $K_{t}$ is gain, H is observer gain, and ${\hat{x}}_{k}^{t}$ is the predicted object state.

This study utilized MATLAB software for dataset creation, model training, and execution. Datasets created in MATLAB software can be stored in the Workspace and immediately accessed for model training and model testing without the need for creating another file. In addition, MATLAB’s built-in Deep Network Designer application enables the construction of networks in the form of block diagrams; compared with the conventional practice of constructing and adjusting networks with text files, this approach is quicker and more intuitive. In this study, the pretrained CSPDarknet-53 framework was selected as the backbone. The parameters used to train the YOLO-v4 stunned red-feathered Taiwan chicken identification model are presented in Table 4. The Initial Learning Rate parameter was set to 0.0001 in this study; when training a YOLO-v4 model, the learning rate can be dynamically adjusted. The Learn Rate Schedule parameter was set to piecewise. The Learn Rate Drop Period was set to 90, and the Learn Rate Drop Factor was set to 0.1. The Mini Batch Size refers to the size of the model training input after each round of training set segmentation. In this study, the Mini Batch Size is 3. To prevent model overfitting, L2 Regularization is used to add an additional item to the loss function to improve the model’s performance and reduce its complexity. In this study, L2 Regularization is set to 0.0005. Max Epochs is the maximum training epoch, with one epoch representing the time required to complete training once with a complete training set. In this study, Max Epochs is set to 120. Shuffle was set to every-epoch, indicating that after every epoch, the training set would be shuffled and entered into the model for training again. Verbose Frequency is the cycle of the output training data and was set to 10, meaning that every 10 Mini Batch Sizes would produce one output to provide insight into the current model training data. The Plots parameter was set to training-progress to enable the real-time monitoring of the validation loss and training loss of the current model training. Finally, the Output Network parameter was set to best-validation, meaning that after all the training epochs were completed, the model with the best validation loss performance would serve as the final output.

**Figure 5:**
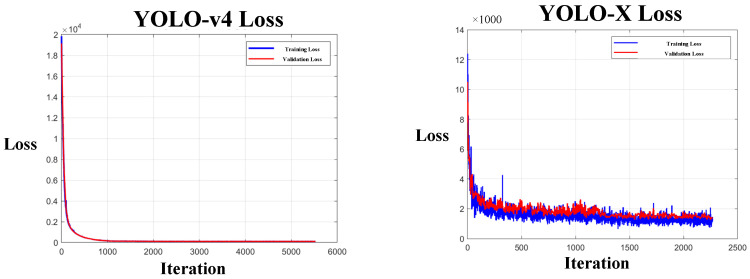
Changes in training loss and validation loss for the YOLO-v4 and YOLO-X models.

The dataset of 1024 labeled photographs comprised 444 images of unstunned red-feathered Taiwan chickens and 580 images of stunned red-feathered Taiwan chickens. This dataset was segmented, at a ratio of 3:1:1, into 614 images in the training set, 205 images in the testing set, and 205 images in the validation set. The YOLO-v4 model trained in this study employs transfer learning, using a pretrained model as the foundation for training the stunned red-feathered Taiwan chicken identification model. This approach was employed to minimize the training duration and number of training iterations by eliminating the need to adjust the initial values of the model. The model training process involved continuous feedback regarding the training loss and validation loss. Training loss can be used to measure the performance of the model training set and reflects the difference between the training data and the actual labels; the lower the output value, the more effective the model. Validation loss reflects the model’s performance in relation to the validation set during the training process. Comparing the validation loss with the training loss can help determine whether the model is overfitted. If the training loss continues to decrease while the validation loss increases, the model is overfitted. In addition, validation loss can reveal the optimal number of epochs for the training set. In this study, the model with the best validation loss within the set number of epochs was chosen as the study’s output model. At the outset of model training, the training loss was 19,843.082, and the validation loss was 19,136.562; both losses then decreased steadily as the number of epochs increased, dropping to 0.105 and 0.105, respectively, by the time the final training was completed. Figure 5 depicts the changes in training loss and validation loss for the YOLO-v4 model and the YOLO-X model. Figure 5 also shows that the YOLO-v4 model for poultry slaughtering recognition in transient stability, in terms of training loss and validation loss, performs better than the YOLO-X model.

### 3.6. Enhancing Images of Stunned Red-Feathered Taiwan Chickens

**Figure 6:**
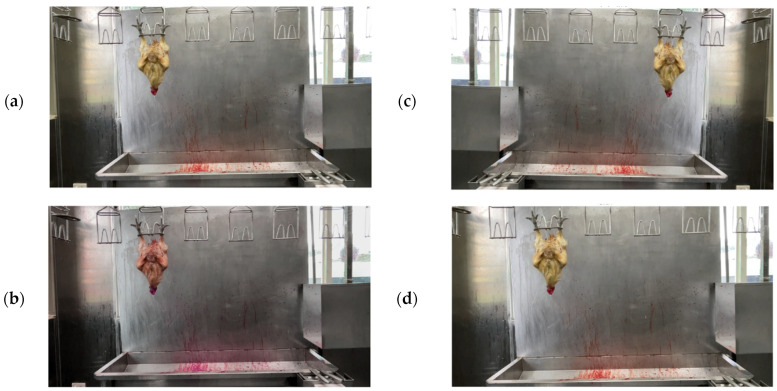
Images captured before and after enhancement techniques: (**a**) original image, (**b**) enhanced image after HSV dithering, (**c**) enhanced image after horizontal flipping, and (**d**) enhanced image after larger scaling by 10%.

After being labeled, the training images were enhanced to increase the variety of the training set. Three image enhancement techniques were performed on the training set. Figure 6a shows the original image of the stunned chicken. The first was HSV color dithering, which adjusts the hue, saturation, and value of the training set images, see Figure 6b. The second technique was horizontal flipping, which enables the model to learn the opposite features of the original image and thus correctly identify when the chicken is convulsing due to a stress response, see Figure 6c. The third technique is larger scaling by 10%, see Figure 6d. The growth of the red-feathered Taiwan chickens in this study may not have been consistent, and such inconsistency may have resulted in varying physical sizes upon their entry to the slaughter line. Random scaling enabled the model to adapt red-feathered Taiwan chickens of varying size. The images in the training set were enhanced to expand it to 3072 images, which were then fed into the model for training.

## 4. Results and Discussion

### 4.1. Performance Criteria of the Stunned Red-Feathered Taiwan Chicken Identification Model

**Figure 7:**
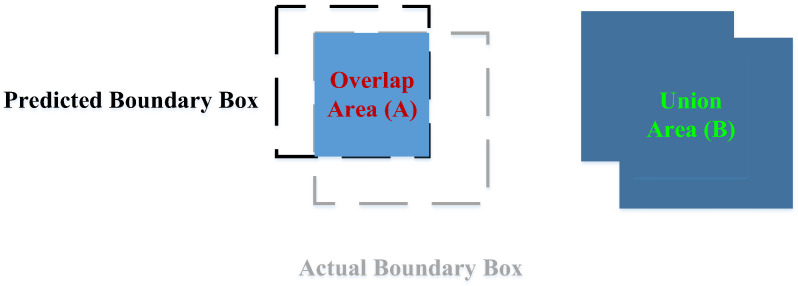
The overlap area (A) and the union area (B) between the predicted boundary box and the actual boundary box.

Metrics, including precision, recall, intersection over union (IoU), average precision (AP), and mean average precision (mAP), were used to evaluate the performance of the red-feathered Taiwan chicken identification model. Figure 7 shows the overlap area (A) and the union area (B) between the predicted boundary box and the actual boundary box.

IoU can be expressed as follows:

(7) $$IoU=\frac{A}{B}$$

where A is the overlap area between the predicted boundary box and the actual boundary box, and B is the union area between the predicted boundary box and the actual boundary box. Evaluating a model on the basis of its IoU requires presetting a threshold; when the IoU is greater than this threshold, the overlap between the prediction result and the actual labeled result is sufficiently high, and the prediction can therefore be considered accurate. Setting an IoU threshold enabled true positives (TPs) and false positives (FPs) to be distinguished. Typically, the IoU threshold is set to 0.5 or higher. In this study, the threshold was set to 0.75. Table 5 illustrates the determination criteria for distinguishing between actual targets and predicted targets of the image recognition model for the red-feathered Taiwan chicken as a confusion matrix.

Precision was defined as the proportion of correct identification among all identified chickens by the model and was calculated using Equation (8):

(8) $$Precision=\frac{TP}{TP+FP}$$

Recall refers to the proportion of correct identification among all predicted targets and was calculated using Equation (9):

(9) $$Recall=\frac{TP}{TP+FN}$$

After calculating the precision and recall, a Precision–Recall curve can be plotted; this curve clearly shows the precision values for various recall rates. In addition, mAP can be expressed as follows:

(10) $$mAP=\frac{\sum_{i=1}^{N}AP_{i}}{N}$$

where N is the number of classification identified targets and i is the category of identified targets. In this study, N=2, inclusive of unconscious and conscious red-feathered Taiwan chickens.

### 4.2. Results of the YOLO-v4 Stunned Red-Feathered Taiwan Chicken Identification Model

**Figure 8:**
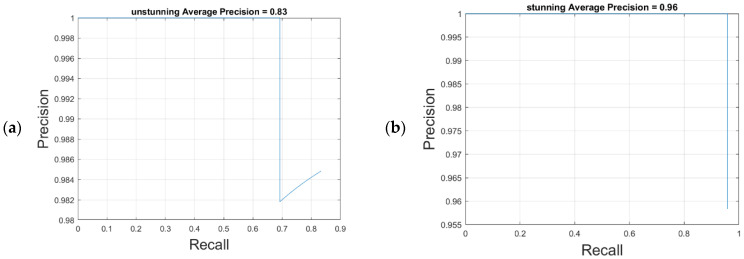
YOLO-v4 model without image amplification: (**a**) Unstunning average precision, (**b**) stunning average precision.

**Figure 9:**
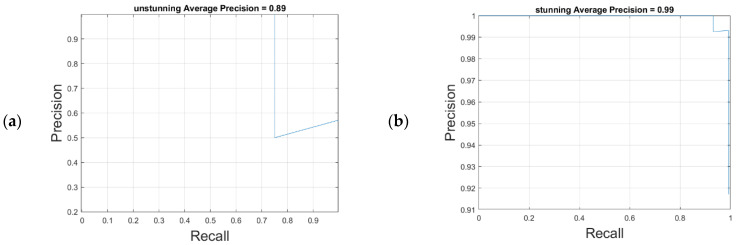
YOLO-v4 model with image amplification: (**a**) Unstunning average precision, (**b**) stunning average precision.

Table 6 shows the confusion matrix of the YOLO-v4 stunned red-feathered Taiwan chicken identification model. As illustrated in Figure 8, with an IoU of 0.75, the YOLO-v4 stunned red-feathered Taiwan chicken identification model without image amplification demonstrated AP scores of 83% for unstunned chickens and 96% for stunned chickens. After the image amplification shown in Figure 9, these scores increased by 6% to 89% for unstunned chickens and by 3% to 99% for stunned chickens; the mAP was 94%, and the image processing speed was 39 frames per second.

The effectiveness of image amplification in enhancing model precision was evident, particularly in improving the detection of unstunned chickens. This enhancement demonstrates the contribution of data augmentation to model generalization and stability.

Evidently, in this study, image amplification of the training set improved the precision of the model. These results verify the effectiveness of image amplification techniques in improving model performance, particularly in the identification of stunned and unstunned red-feathered Taiwan chickens. The application of image amplification techniques in model training significantly improved the accuracy of the YOLO-v4 model, enhancing its reliability and stability in practical applications.

**Figure 10:**
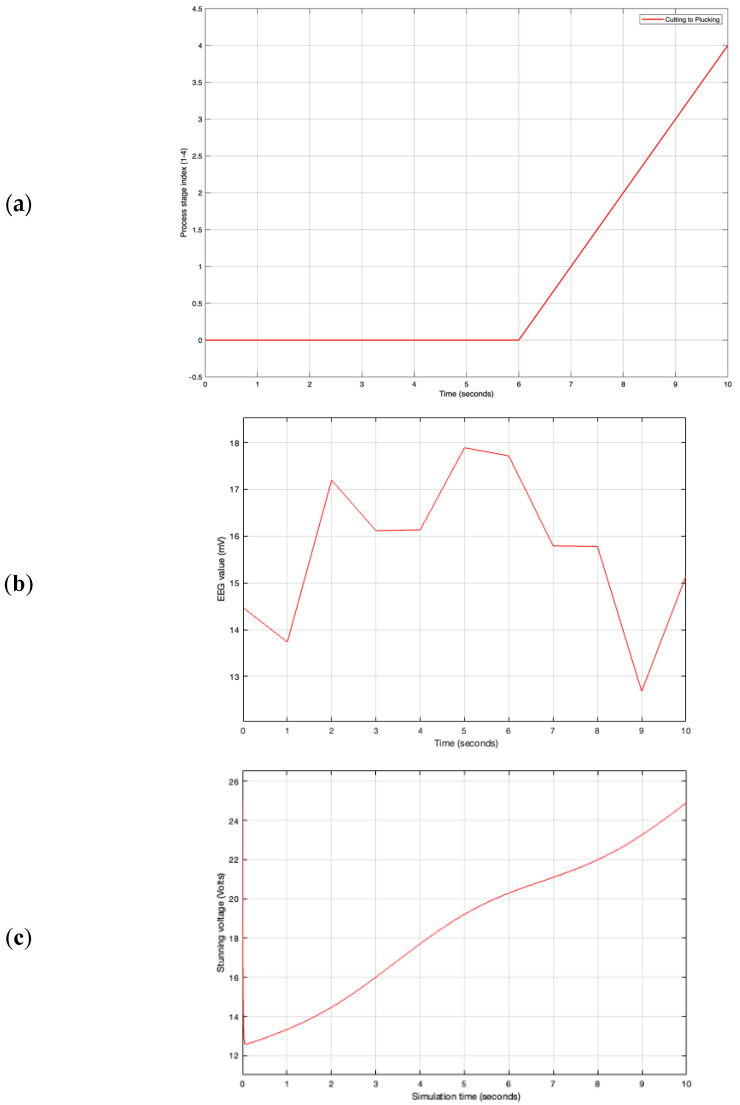
(**a**) Process stage progression output from virtual conveyor simulation, (**b**) EEG signal time series and threshold evaluation results, and (**c**) stunning voltage output from PID controller (Voltage vs. Time).

The virtual conveyor simulation (Figure 2a) successfully replicated the stage-wise progression of poultry through the slaughtering process. Over a 10 s simulation period, transitions from idle to reception, stunning, cutting, and plucking were accurately modeled. The stage index signal output (Figure 10a) confirmed correct sequencing, with the index values increasing from 0 to 4 in alignment with real-time process logic. These results validate the model’s ability to capture and represent synchronized physical stages in accordance with digital twin principles.

The EEG-based stunning detection subsystem (Figure 2b) employed a 12 mV threshold to evaluate the consciousness state of the chickens. As illustrated in Figure 10b, the simulated EEG signal fluctuated within the range of 8–20 mV but consistently remained above the threshold. Consequently, the system continuously classified the subject as “not stunned”. This outcome demonstrates the reliability and responsiveness of the classification logic, supporting its application in sensor-driven, IoT-enabled animal welfare monitoring frameworks.

Figure 2c shows the PID-based voltage control structure, which was developed to simulate adaptive voltage regulation in response to real-time weight variations. The corresponding output (Figure 10c) shows a steady increase in stunning voltage from approximately 13 V to 26 V, reflecting the controller’s effective regulation across the simulation window. These findings support the feasibility of integrating real-time closed-loop control mechanisms within smart stunning systems designed for IoT-based poultry processing environments.

**Figure 11:**
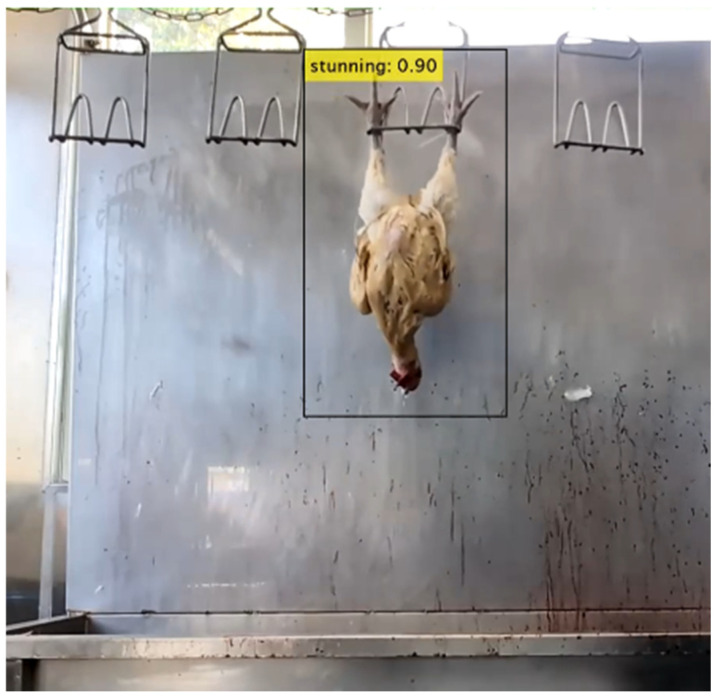
Dynamic tracking object recognition in a real environment.

Finally, the model was deployed and tested in a real slaughterhouse environment. Figure 11 illustrates the dynamic tracking object recognition for the red-feathered Taiwan chicken in a real slaughterhouse environment. In this figure, the stunned accuracy is 0.90. This confirms its robustness and suitability for real-time object tracking applications in industrial poultry processing.

## 5. Conclusions

This study successfully built a smart and humane poultry slaughtering system to monitor and manage poultry on the slaughter line. By integrating cameras, the system enables the real-time tracking of poultry status, significantly reducing the need for manual inspection. A YOLO-v4-based deep learning model was trained to recognize red-feathered Taiwan chickens using firsthand data from the slaughterhouse, achieving 99% and 89% accuracies in detecting stunned and unstunned chickens, with a mean average precision (mAP) of 94% at IoU thresholds of 0.75 and an image processing speed of 39 frames per second. The system ensures humane slaughter practices and can be widely applied in modern poultry processing facilities and other chicken breeds to support animal welfare, hygiene standards, and automation. The inclusion of EEG-based stunning detection, PID voltage control, and real-time simulation modeling lays the groundwork for IoT-enabled remote management, predictive monitoring, and system scalability. Furthermore, in terms of transient stability, the YOLO-v4 model for poultry slaughtering recognition performed better than the YOLO-X model with respect to training loss and validation loss in this study. Overall, this smart slaughtering system represents a practical and scalable application of AI in the poultry industry.

## Figures and Tables

**Table 1:** System equipment specifications.

Equipment	Brand	Model	Specification
CPU	Intel, USA	i7-14700K	Cores: 20 Threads: 28
Graphics Processing Unit (GPU)	NVDIA, USA	RTX 4080 Super	Memory: 16 GB, GDDR6X Memory speed: 23 Gbps Memory bandwidth: 736 GB/s Base clock rate: 2295 MHz
Random Access Memory (RAM)	Kingston, Taiwan	DDR5-5600	32 GB
Hard Drive	Western Digital, China	SN770 SSD	1 TB
Operating System	Microsoft, USA	Windows 11	Business Editions
Wi-Fi Router	D-Link, Taiwan	DWR-953	Integrated SIM card slot 4G LTE Fail-safe Internet with fixed line and mobile Internet support
Camera A	Xiaomi, China	AW200	Resolution: 1920 × 1080 Camera angles: 120° Memory: 256 GB
Camera B	Xiaomi, China	C300	Resolution: 2304 × 1296 Lens movement: 360° horizontally, 108° vertically Memory: 256 GB

**Table 2:** Brainwave data sheets of electric shocks administered to red-feathered Taiwan chickens.

Scenario	Total Brainwave Energy Before Stunning	Post-Stunning Criterion of Unconsciousness	P1	P2	P3
			Brainwave Energy	Brainwave Energy	Brainwave Energy
1	4.81	0.48	0.03	0.04	0.07
2	3.22	0.32	0.04	0.15	0.06
3	6.23	0.62	3.05	2.11	3.05
4	2.77	0.28	5.58	6.95	7.15

**Table 3:** Visual judgements of red-feathered Taiwan chickens.

Raise Head	Open Eyes	Move Wing	Weight	Voltage
No	No	No	2.33 kg	160 V
No	Yes	No	3.01 kg	100 V
Yes	Yes	No	2.84 kg	80 V
No	No	No	2.82 kg	100 V

**Table 4:** Training parameters of the YOLO-v4 stunned red-feathered Taiwan chicken identification model.

Parameter	Settings
Input Size	[608×608×3]
Gradient Decay Factor	0.9
Squared Gradient Decay Factor	0.999
Initial Learn Rate	0.0001
Learn Rate Schedule	Piecewise
Learn Rate Drop Period	90
Learn Rate Drop Factor	0.1
Mini Batch Size	3
L2 Regularization	0.0005
Max Epochs	120
Shuffle	every-epoch
Verbose Frequency	10
Plots	training-progress
Output Network	best-validation

**Table 5:** The determination criteria for distinguishing between actual targets and predicted targets of the image recognition model for red-feathered Taiwan chickens.

Determination Result	Actual Target	Predicted Target
TP_1	Stunned chicken	Stunned chicken
TP_2	Unstunned chicken	Unstunned chicken
FP_1	Unstunned chicken	Stunned chicken
FP_2	Stunned chicken	Unstunned chicken
FP_3	Background	Stunned chicken
FP_4	Background	Unstunned chicken
FN_1	Stunned chicken	Background
FN_2	Unstunned chicken	Background
FN_3	Background	Stunned chicken
FN_4	Background	Unstunned chicken
TN	Background	Background

**Table 6:** The confusion matrix of the YOLO-v4 stunned red-feathered Taiwan chicken identification model.

YOLO-v4 IoU = 0.75	Actual Stunned Chicken	Actual Unstunned Chicken	Actual Background
Predicted stunned chicken	TP_1 = 104	FP_1 = 1	FN_3 = 0
Predicted unstunned chicken	FP_2 = 1	TP_2 = 99	FN_4 = 0
Predicted background	FN_1 = 0	FN_2 = 0	TN = 0

## Data Availability

The original contributions presented in this study are included in the article. Further inquiries can be directed to the corresponding author.
